# Shaped electrodes for adaptive X-ray optics

**DOI:** 10.1107/S1600577526003747

**Published:** 2026-05-07

**Authors:** Kenneth A. Goldberg, Francesco Marzari, Grant D. Cutler

**Affiliations:** a Advanced Light Source Lawrence Berkeley National Laboratory Berkeley CA94720 USA; b Department of Physics University of Trento via Sommarive 14 38123Trento Italy; c National Institute for Nuclear Physics (INFN) via Sommarive 14 38123Trento Italy; d Department of Industrial Engineering University of Trento via Sommarive 9 38123Trento Italy; DESY, Germany

**Keywords:** adaptive optics, lithium niobate, X-ray mirror, wavefront, focusing

## Abstract

Lithium niobate has emerged as a promising substrate material for adaptive X-ray mirrors. Heat treatment induces ferroelectric domain inversion, yielding two oppositely poled, layered regions that enable lithium niobate to behave as a monolithic bimorph in the presence of an applied electric field. In addition to fabrication simplicity, this well known material has favorable actuation and shape-control properties without hysteresis, creep, and drift. Following Timoshenko’s approach to the bi-metallic strip thermostat, we derive an analytic description of shaping continuous electrode widths to produce a spatially varying surface curvature when a voltage is applied. Through comparison with finite-element analysis, the method is demonstrated for uniform electrode widths that produce cylindrical surfaces, and for varying electrode widths that produce plane-parabolic and plane-elliptical surfaces from initially flat substrates. A proposed longitudinally continuous, compound electrode pattern shows how a single flat mirror could be shaped to achieve multiple profiles with a single applied voltage.

## Abstract 2

For adaptive X-ray optics on high-performance beamline optical systems, we describe an approach to mirror figure and slope control using patterned, shaped electrodes to control the local curvature on a lithium niobate substrate from a single applied voltage. Longitudinally continuous electrode patterns achieve target shapes without discontinuities, and compound electrodes enable multiple target shapes to be achieved on a single substrate.

## 1. Introduction

X-ray mirrors on beamlines at modern synchrotron light sources and free-electron lasers require surface shape and slope specifications on the order of nanometres and tens of nanoradians to reach their ultimate performance goals (Church & Takacs, 1993 ▸; Shi *et al.*, 2016 ▸; Cocco *et al.*, 2022 ▸). For high-coherent-flux beamlines especially, interest in adaptive X-ray optics (AXO)—mirrors with dynamic shape-changing ability—has increased as the technology has become more mature. Such mirrors offer wavefront correction or control at unprecedented levels.

Highly polished X-ray mirrors are reflective at small, glancing angles of incidence (typically below 3°), making the beam footprint long and narrow. Shape actuation is most effective in the longitudinal (tangential) direction, where small changes can have a significant impact on the reflected wavefront.

Several types of adaptive mirrors have been developed and tested in the past four decades. Mirrors can be bent from flat or prefigured with a desired shape and actuated for fine control. Mechanical benders are commonly used to provide second-order and third-order shape control (Howells, 1995 ▸; Yashchuk *et al.*, 2013 ▸; Nistea *et al.*, 2017 ▸). Early demonstrations of adaptive X-ray mirrors with arrays of addressable channels (Susini *et al.*, 1995 ▸; Susini *et al.*, 1996 ▸; Signorato *et al.*, 1998 ▸) paved the way for numerous refinements (Sawhney *et al.*, 2010 ▸; Mimura *et al.*, 2010 ▸; Alcock *et al.*, 2015 ▸; Ichii *et al.*, 2019 ▸; Inoue *et al.*, 2024 ▸), including high speed (Nistea *et al.*, 2025 ▸) and nm-level precision (Nistea *et al.*, 2025 ▸; Gunjala *et al.*, 2023 ▸). Hybrid designs with mechanical bending for large changes and piezoceramic actuators for fine control have also been created (Inoue *et al.*, 2021 ▸).

Recent demonstrations of adaptive mirror bending with lithium niobate (LN) mirror substrates have sparked great interest for several important reasons (Inoue *et al.*, 2024 ▸; Inoue *et al.*, 2025 ▸). Heat treatment produces two oppositely poled ferroelectric domains, an inherently piezoelectric layered structure that behaves like a monolithic piezoelectric bimorph (Nakamura *et al.*, 1987*a* ▸). Although the transverse piezoelectric strain coefficients are close to 20 times smaller in LN than in lead zirconate titanate (PZT) materials, lithium niobate shows no hysteresis or drift, simplifying shape control. Using LN thus eliminates the need for bonded, glued, or deposited piezoceramic materials, which are commonly added to inert substrates. Where possible, the top surface electrode can double as the reflective coating.

Lithium niobate exhibits a strong asymmetry in piezoelectric response depending on the crystal orientation. The rotated *Y*-cut at 74.3° is an orientation that enables unidirectional expansion which is ideal for adaptive mirror bending. The *d*_21_ coupling constant is −6.6453 × 10^−12^ C N^−1^, equivalent to −6.6453 pm V^−1^ (Marzari *et al.*, 2026 ▸). Other orientations offer stronger coupling constants, at the expense of sagittal curvature. The creation of two domains with opposite polarization inside lithium niobate requires spontaneous polarization to be non-constant through the thickness of the substrate: rotated *Y*-cut plates with inclined spontaneous polarization show ferroelectric domain inversion caused by heat treatment (Nakamura *et al.*, 1987*b* ▸).

In general, the induced curvature increases with the applied voltage and decreases with the square of the substrate thickness (Susini *et al.*, 1995 ▸; Goldberg & La Fleche, 2023 ▸). Although shape control through thickness variation has been described (Tian *et al.*, 2024 ▸), most applications have used uniform substrate thickness.

To achieve fixed, pre-determined bending shapes, we propose longitudinally continuous electrodes patterned with a varying sagittal width on a uniformly wide substrate. Such electrodes span the length of the mirror in the tangential direction. Upon actuation, the local curvature varies as a linear function of the electrode width and the applied voltage. Lithographic patterning can be applied to control the electrode shape to sub-micrometer accuracy. Continuous electrodes eliminate the so-called *junction effect* which is observed at discontinuities or gaps in segmented electrode arrays in some mirror designs (Alcock *et al.*, 2013 ▸). We derive the functional dependencies and show how continuous electrodes could be used to achieve predefined surface shapes.

## 2. Derivation

The derivation follows Timoshenko’s well known study of the thermal dependence of a bi-metallic strip (Timoshenko, 1925 ▸), wherein two materials with different thermal expansion coefficients are bonded along their length. Temperature changes induce a curvature in the system.

Piezoelectric bimorph systems operate by a closely related mechanical principle: an applied electric field takes the place of the temperature change (Susini *et al.*, 1995 ▸; Conrad *et al.*, 2008 ▸). Selected piezoelectric materials expand in the direction orthogonal to the applied field. Heat-treated lithium niobate exhibits similar behavior from an inherent separation of layers with opposite polarization occurring in the thickness direction (Nakamura *et al.*, 1987*a* ▸; Nakamura & Shimizu, 1989 ▸). With an applied electric field, the strain is parallel to the layer boundary. The charge-coupling parameters have opposite sign in the two layers, enabling a single applied field to generate the differential strain that bends the material.

**Figure 1:**
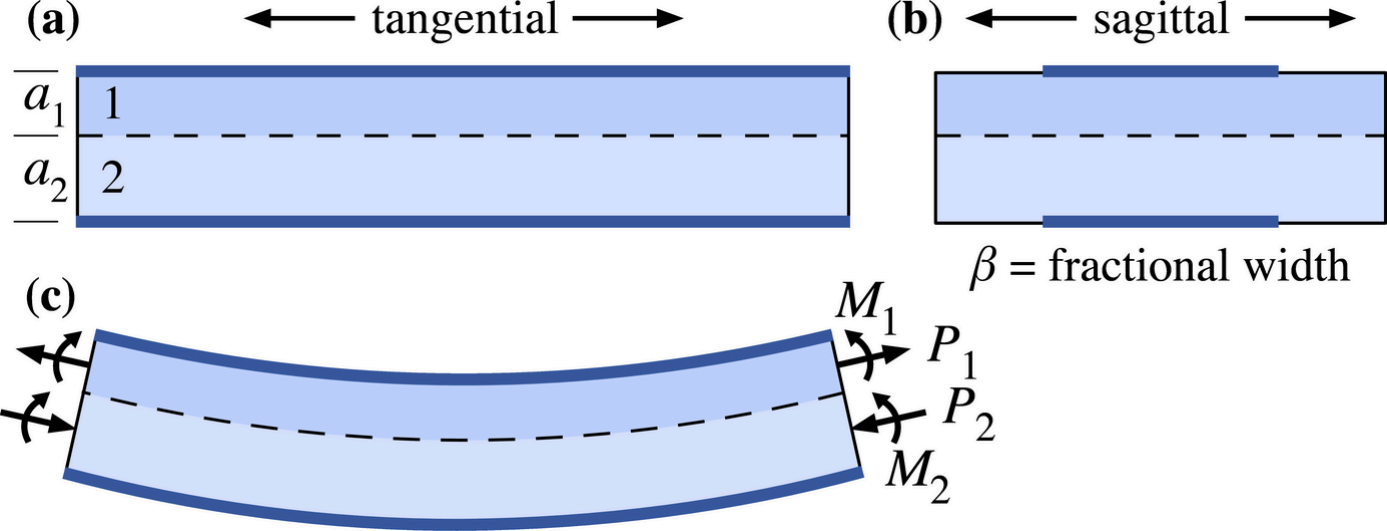
Deflection of a lithium niobate X-ray mirror substrate, shown in detail. Surface electrodes do not span the sagittal width of the mirror: β is the fractional electrode width.

In our approach, the substrate has uniform width and matching, patterned electrodes on the top and bottom sides (see Fig. 1 ▸.) The electrode widths vary along the tangential direction of the mirror. For simplicity, we assume the electric field runs uniformly between the electrodes, much like a capacitor—fringe fields are neglected. In this approximation, the induced layer expansion is proportional to the fractional width of the electrodes along the substrate. Two phases of the LN material are present, occupying uniform layer thicknesses of *a*_1_ and *a*_2_, with a total thickness of *h* = *a*_1_ + *a*_2_. Each layer is treated as homogeneous.

Following Euler–Bernoulli beam theory, we assume that plane cross-sections remain perpendicular to the neutral axis. All of the forces acting on the concave side of layer 1 can be represented by an axial tensile or compressive force, *P*_1_, and bending moment, *M*_1_. Similarly, the convex side of layer 2 has *P*_2_ and *M*_2_. In equilibrium, in the absence of external forces, we have 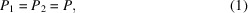

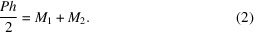
Let ρ be the local radius of curvature. We assume ρ ≫ *h*. In the case of LN, the two layers are made of the same material. Thus, we take *E*_1_ = *E*_2_ = *E* as Young’s modulus of elasticity. Since the layer thicknesses can be different, we have separate *I*_1_ and *I*_2_, the area moments of inertia. The flexural rigidity of the respective layers are *EI*_1_ and *EI*_2_, 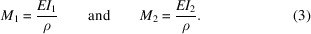
Thus, with substitution, 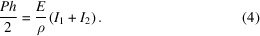
Solving for *P*, 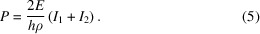
Following Conrad *et al.* (2008 ▸), Timoshenko’s thermal strain is replaced by the equivalent piezoelectric property: α_*i*_Δ*T* becomes β*d*_21_*U*/*h*. With *U* as the applied voltage, the electric field in the layers is *U*/*h*. Assuming the electric field is uniform between the top and bottom electrodes and falls to zero in the adjacent regions, the parameter β scales the layer expansion forces by the fractional width of the actuator, from 0 to 1. To create concave curvature, layer 1 contracts, while layer 2 expands.

Longitudinal elongation along the interface must be equal,
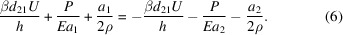
Gathering terms, 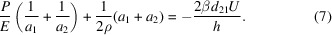
We substitute *P* from equation (5), and take *h* = *a*_1_ + *a*_2_ where possible,
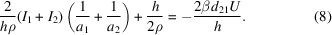
With unit width, the area moments of inertia are *I*_1_ = 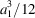 and *I*_2_ = 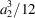. Multiplying through by *h*ρ, 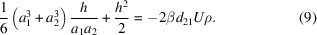
Factoring the third-order polynomial on the left-hand side and combining like terms leads to a curvature, κ, 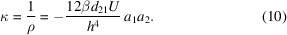
The dependence on the layer thicknesses becomes clear if we define a dimensionless parameter *t* to represent the fractional thickness of layer 1: with *t* ∈ [0, 1], *a*_1_ = *th* and *a*_2_ = (1 − *t*)*h*. Then, 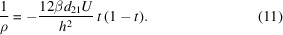
The curvature has linear dependence on the fractional electrode width β, the applied voltage *U*, and the strengths of the coupling constant, *d*_21_. It is inversely related to the square of the total thickness. The surface becomes flat as one or the other layer thickness tends toward zero: *t* → 0 or *t* → 1.

In the case of equal layer thicknesses (*t* = 1/2) we have a simple expression that we will use in the proceeding analysis,
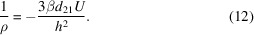


## 3. Shaped electrodes

Designing β to vary along the length of the mirror presents interesting opportunities for surface shape control. For a given target mirror shape with surface height *z*(*y*), varying in the tangential, *y* direction, the required fractional electrode width β(*y*) can be determined as follows.

The local curvature is connected to the first and second derivatives of *z*(*y*) by the curvature formula,
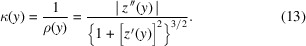
In X-ray optics, the slope *z*′ typically stays close to zero, and the curvature is closely approximated by the second derivative, 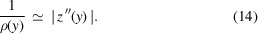
(At 1° slope, the difference between κ and *z′′* is below 0.05%.) Removing the absolute value, we can assign ρ > 0 for concave surfaces and ρ < 0 for convex.

Solving equation (10) for β(*y*) at all points along the mirror’s length, we find a solution with arbitrary layer thicknesses, with positive, concave curvature: 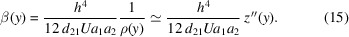
In the equal-layer-thickness case, equation (15) simplifies to 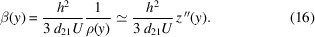


### 3.1. Selecting the applied voltage

Designing a variable-width electrode requires the selection of a voltage that allows β(*x*) to span a useful range. Suppose the minimum radius of curvature is ρ_min_. This is the maximum curvature condition. Since β cannot exceed 1, the minimum useful value for *U*, from equation (15), is 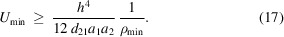
(Note that convex curvatures require attention to the voltage polarity here.)

When the electrode serves as the mirror’s reflective coating, it must be wider than the beam’s width, *w*. Thus, for a mirror of width *W*, 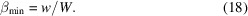
At the minimum β_min_, an upper limit on the applied voltage now comes from the maximum radius of curvature, ρ_max_, 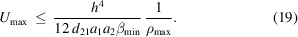


## 4. Modeling uniform electrode widths

**Figure 2:**
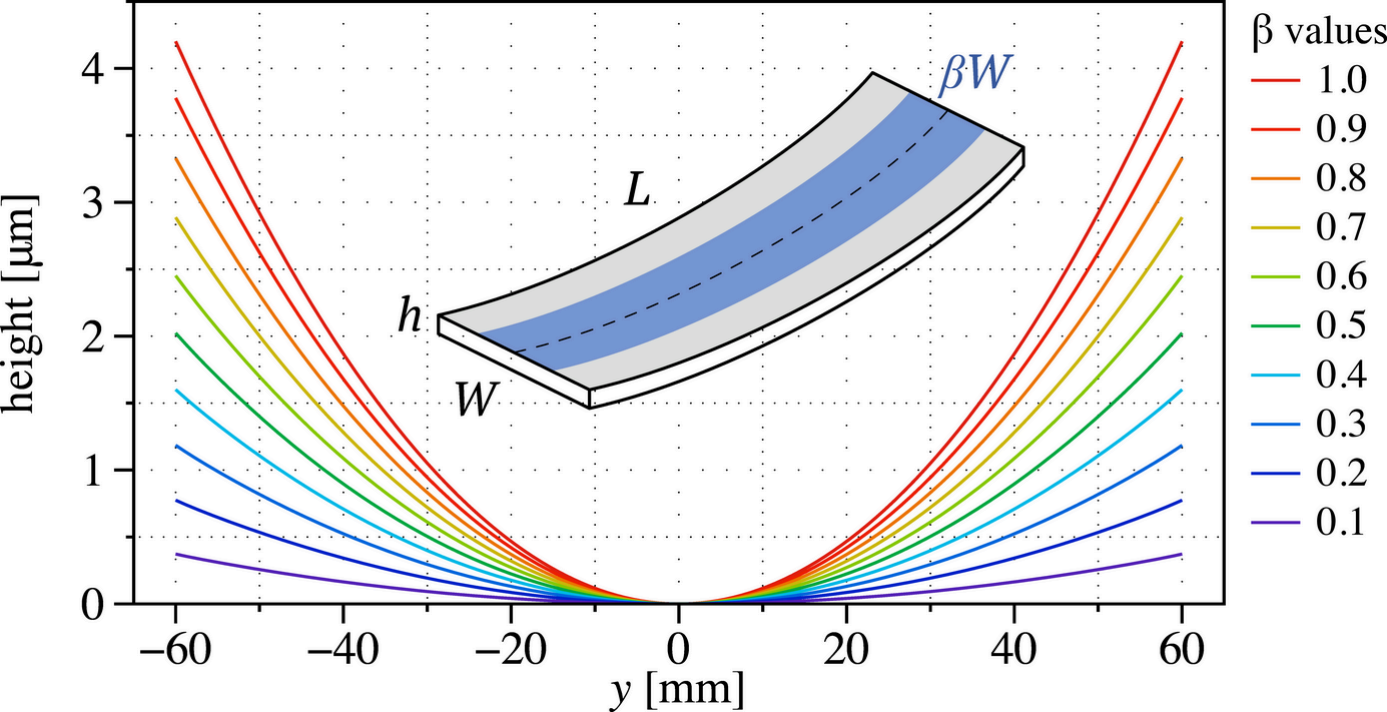
Surface height along the central meridian, modeled with finite-element analysis (FEA) for ten different uniform electrode widths. The fractional width, β, varies from 0.1 to 1.0.

As a check of our analytical model, we calculate the actuated shape of the mirror for a range of electrode widths using the finite element method. Our finite element model is implemented in the *ANSYS* software (Ansys, 2025 ▸) using coupled-field elements (ANSYS SOLID226). The meshed geometry is a rectangular strip *L* = 120 mm long, *W* = 20 mm wide, and *h* = 0.5 mm thick. (See the inset image in Fig. 2 ▸.) Anisotropic material properties for the 74.3° *Y*-cut orientation of LN are calculated and the matrices are constructed using the process described by Marzari *et al.* (2026 ▸). The electrical boundary conditions are imposed by setting the top electrode to +*U* and grounding the bottom electrode. Structural boundary conditions are applied to nodes on the planes of symmetry; nodal displacement degrees of freedom perpendicular to the plane are fixed, corresponding to an ideal strain-free support system.

**Figure 3:**
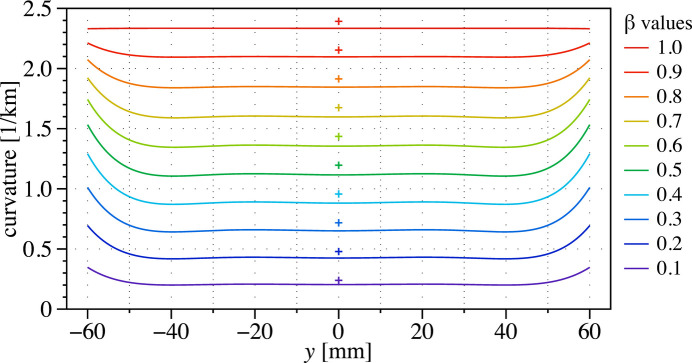
FEA modeled curvature calculated from the ten surfaces in Fig. 2 ▸. Crosses indicate the constant curvature expected from equation (12).

**Figure 4:**
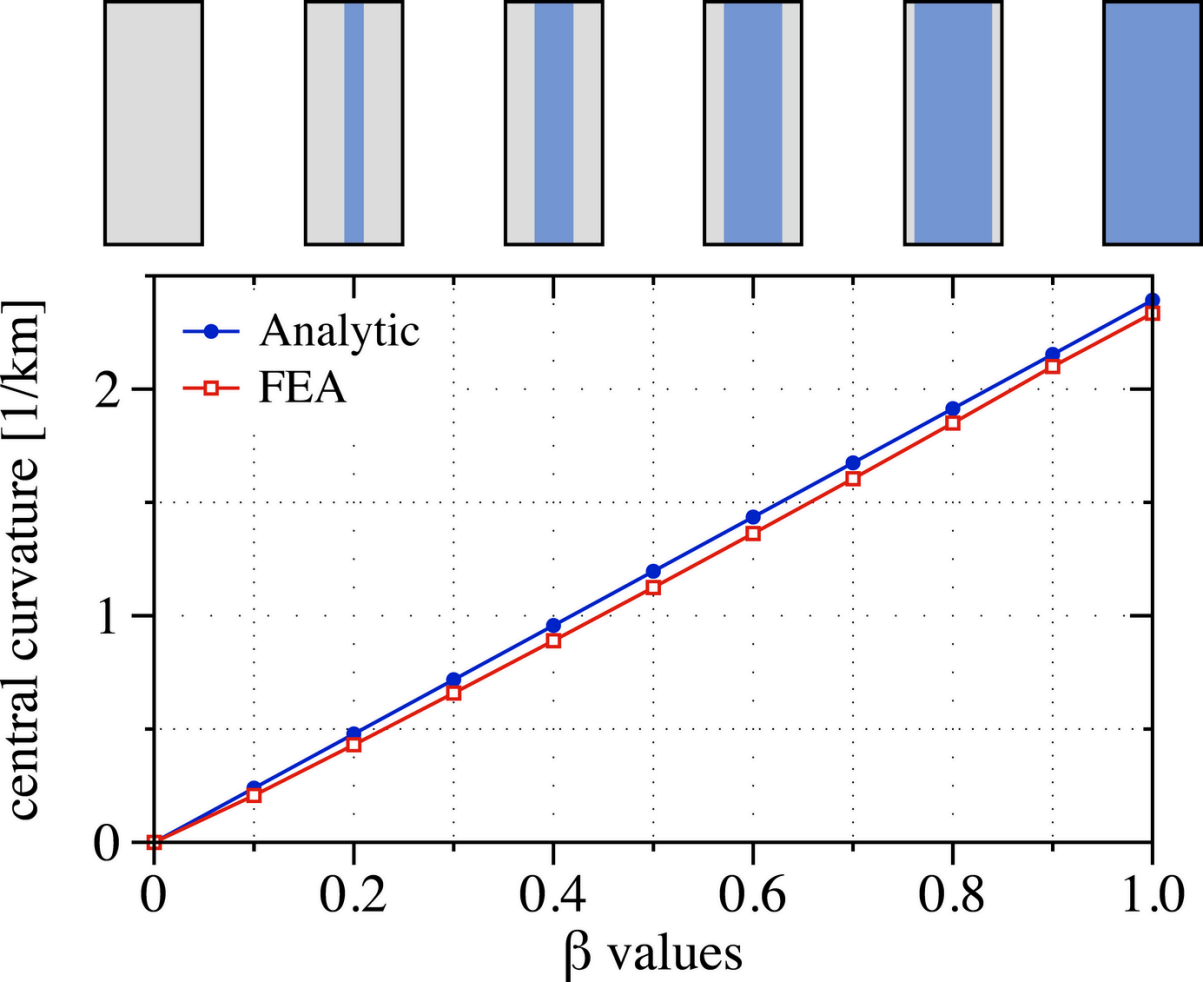
Comparison of the central curvature derived analytically [equation (12)] and the FEA-modeled surfaces from Figs. 2 ▸ and 3 ▸. Schematic representations of the electrode coverage are shown above the plot, not to scale.

We modeled electrode widths in ten even steps from β = 0.1 to 1.0. Fig. 2 ▸ shows the resultant, approximately cylindrical surface height profiles along the central meridian. The curvature of each profile, along the mirror’s length, is shown in Fig. 3 ▸. For comparison, the curvatures predicted by equation (12) are shown as crosses in Fig. 3 ▸ and for all β values in Fig. 4 ▸. The curvature dependence on β is nearly linear, but an over-prediction of as much as 2% is apparent.

## 5. Case studies

We consider an application of this approach to mirrors bent from flat into plane-parabolic and plane-elliptical surface shapes. These configurations match the final vertical focusing mirror on a planned, hard X-ray scattering beamline at the Advanced Light Source. The mirror is designed with a fixed source conjugate distance, *p* = 3.5 m, a central angle of incidence, θ = 0.2°, and three different focusing conditions. Case 1 vertically collimates the diverging beam with a plane-parabolic shape, *q*_1_ = ∞. Case 2 focuses to a detector plane, *q*_2_ = 2.50 m. Case 3 focuses to a sample position, *q*_3_ = 1.45 m.

The parabolic (Goldberg, 2022*a* ▸) and elliptical (Goldberg, 2022*b* ▸) mirror surface shapes were calculated from closed-form expressions, derived in the mirror-centered coordinate system, with zero slope at the origin. (Note that the parabolic shape matches an elliptical shape with an infinite conjugate distance.)

**Figure 5:**
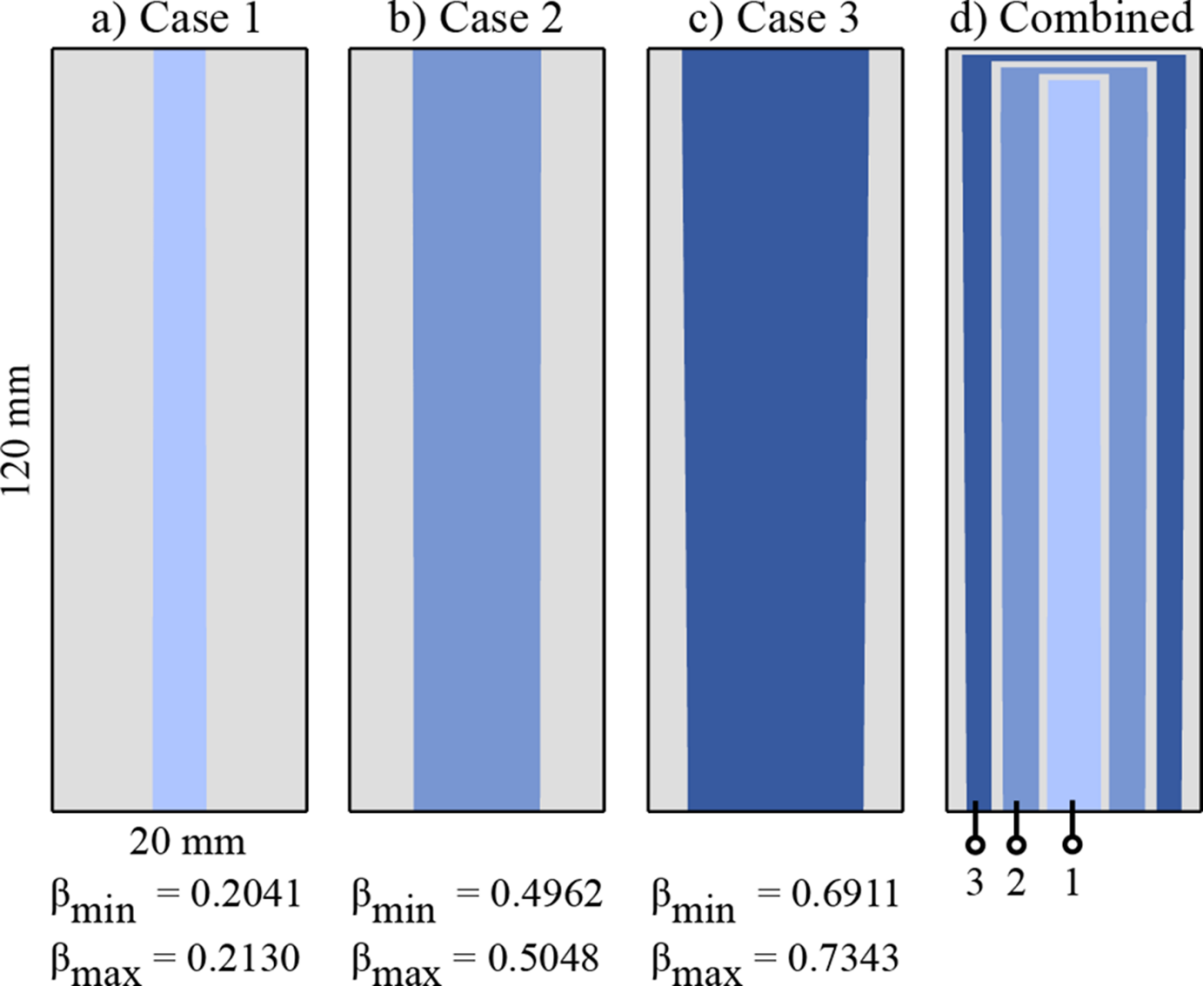
Shaped electrode profiles for the three cases are shown as blue regions in (*a*), (*b*), and (*c*). The length dimension has been compressed for visualization. A compound electrode shown in (*d*) allows a single mirror to achieve three surface shapes by connecting the voltage to one, two, or all three electrodes.

Consistent with Section 4, we model an LN substrate with length *L* = 120 mm, width *W* = 20 mm, and thickness *h* = 0.5 mm, assuming that the two layers have equal thicknesses. Using the local curvatures calculated from the three surface shapes, equation (16) provides the shaped electrode width profiles. A common, single applied voltage of *U* = 30 V was selected, satisfying equations (17) and (19) for all three target shapes. The calculated electrode profiles used in the finite element analysis (FEA) modeling are shown in Figs. 5 ▸(*a*), 5(*b*) and 5(*c*) with the corresponding range of β values below each.

**Figure 6:**
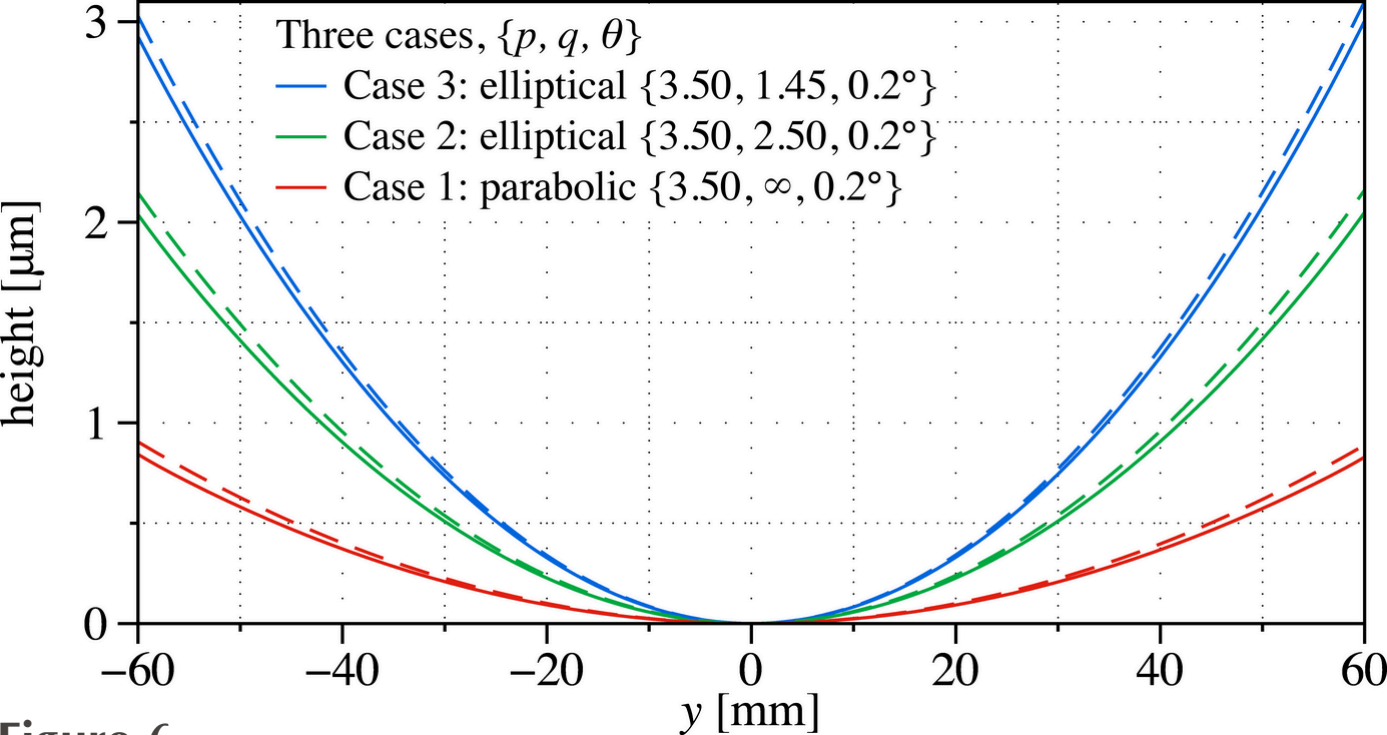
Surface height profiles extracted from the FEA models (solid lines) are compared with the target shapes, calculated analytically (dashed lines). The *p* and *q* distances are given in meters.

Qualitative agreement of the modeled heights and curvatures is apparent in Figs. 6 ▸ and 7 ▸, but we find the FEA-modeled curvature to be consistently less than predicted from the analytic model. This slight under-prediction matches our expectations from the uniform-width electrode models in Section 4. The measured curvature is typically a few percent below the value calculated from equation (12). The difference may come from fringe fields that extend laterally beyond the electrodes and increase the apparent width by a small amount. Increasing the accuracy of curvatures achieved with this approach may require either 3D, fully coupled, structural-electrical models; an iterative, empirical approach performed with feedback from FEA or experimental data; or an electric field model that includes the fringe fields, either analytically or empirically.

The FEA-modeled data in Fig. 3 ▸ predict a curvature deviation near the longitudinal ends of the mirror with a characteristic length of approximately 15 mm. Reducing this effect requires either a tapering of the electrode width at the ends or restricting the beam footprint to a clear aperture that excludes these end regions.

### 5.1. Compound shaped electrodes

Fig. 5 ▸(*d*) shows a compound electrode arrangement containing all three shaped-electrode profiles, nested symmetrically on a single substrate. At each longitudinal position, the combined width of one, two, or all three electrodes matches the widths of Cases 1, 2, and 3 from Figs. 5 ▸(*a*), 5 ▸(*b*), and 5(*c*). The Case 1 surface shape is achieved with voltage applied only to the central channel 1. Case 2 requires equal voltage to channels 1 and 2, as indicated. Case 3 has voltage applied to all three channels. In this way, the compound electrodes achieve bending that matches the three individual-electrode cases.

**Figure 8:**
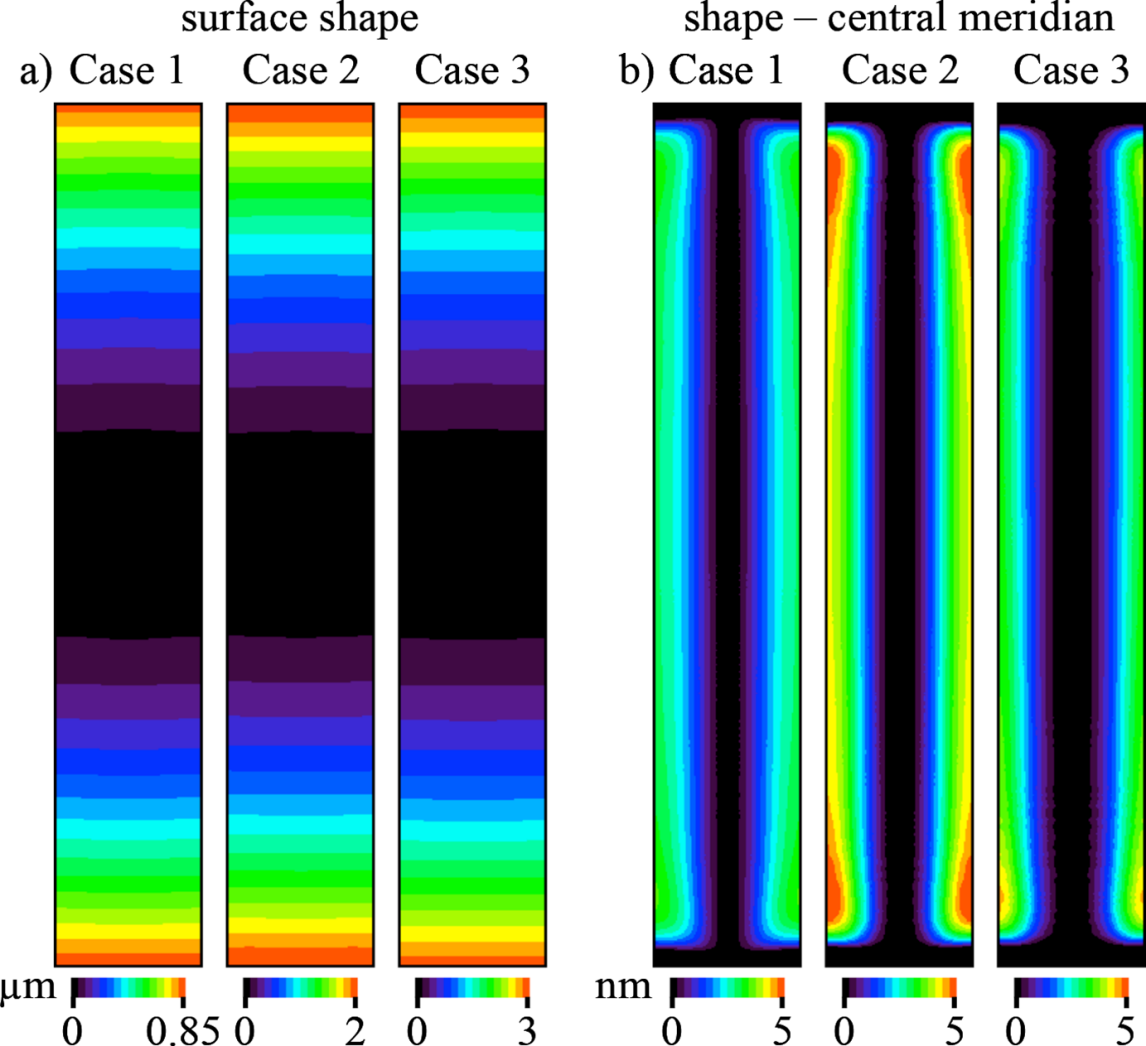
(*a*) Actuated surface shapes from the compound electrodes, spanning the 20 mm × 120 mm area. (*b*) The sagittal curvatures are isolated by subtracting the shape along the central meridian in each case.

Fig. 8 ▸ shows the modeled surface shapes across the full mirror area. The sagittal curvatures and edge effects are revealed [Fig. 8 ▸(*b*)] by subtracting the tangential shape of the central meridian. Fitting the surface shape from a 4 mm-long lateral cross section through the center of the mirror, we find the sagittal radii of curvature in the three cases are 4.456 km, 7.098 km, and 13.354 km, respectively. Although the sagittal curvature is small in all three cases, it is 3.0 times higher in Case 1 than in Case 3—wider electrodes are associated with reduced sagittal curvature.

### 5.2. Electrode width variation

In many possible X-ray beamline applications of this approach, the electrode width variation along the length of the mirror will be modest, especially when the mirror length is a small fraction of the conjugate distances. In geometrical optics, Coddington’s equations (Kingslake, 1994 ▸) describe the local, tangential (meridional) mirror surface curvature to focus light from conjugate distances *p* and *q*. This local curvature is 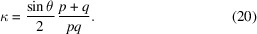
With a plane-elliptical mirror, for example, the conjugate distances and the curvature change along the mirror surface. Relative to the center of the mirror, we approximate *p*(*y*) = *p*_0_ + *y* and *q*(*y*) = *q*_0_ − *y*, and simplify the discussion using a uniform angle of incidence, θ,
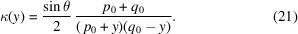
With the central curvature, κ(0), we find for modest mirror lengths, 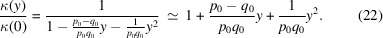
At the extreme ends of the mirror, *y* = ±*L*/2. The fractional variation in curvature is 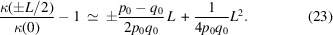
When *L* is much smaller than *p*_0_ or *q*_0_, the linear term in *L* dominates. In the demagnifying focusing condition, when *q*_0_ < *p*_0_, the curvature will reduce (and the electrode width will narrow) from the upstream end to the downstream end of the mirror. Conversely, if the mirror is used for collimating or focusing to a distance *q*_0_ > *p*_0_, then the curvature and the electrode width will increase from upstream to downstream.

In the three cases studied in this section, the electrode width varies by 4.2%, 1.7%, and 5.9%, respectively. Note that when *p* ≃ *q*, the linear term in equation (23) tends to zero, and the curvature variation (along with the electrode width) is second-order, being narrowest at the center.

## 6. Sensitivity analysis

To meet slope- and shape-control requirements at a level of, for example, 100 nrad and 3 nm requires stability and careful engineering. Setting aside the mechanical mounting and power management, we can explore the design and control sensitivities that shaped-electrode mirrors would require. Although mirrors are not typically specified to achieve curvature tolerances, the various degrees of freedom we have discussed herein all affect the curvature. We therefore consider the most challenging case among the shaped electrodes in Section 5, Case 3, and apply several simplifications to understand approximate dependencies.

With a central curvature κ given by equation (12), the slope of our concave mirror, *s*, varies approximately as *s* = κ*y*, and the surface height varies as *z* = 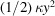. Both functions grow in magnitude with the distance from the center. Achieving a given slope tolerance Δ*S* at position *Y* bounds the allowable, relative change in curvature to 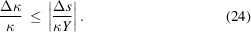
Similarly, the allowable change in κ that preserves a given height change Δ*z* at position *Y* is 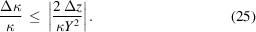
In Case 3, the central curvature is κ = 1.702 × 10^−3^ m^−1^ (ρ = 587.4 m) and β = 0.7114. At the position *Y* = 50 mm, equations (24) and (25) show that the allowable fractional changes in κ are 1.175 × 10^−3^ for the slope requirement, and 1.4 × 10^−3^ to meet the height requirement. We consider the former, tighter specification.

From the first partial derivatives of equation (12), we can estimate dependencies on each parameter. Where β is 0.7114 and *W* = 20 mm, the allowable uniform variation in the electrode width is 16.72 µm. At *U* = 30 V, the allowable variation is 35.25 mV. At *h* = 0.5 mm, the allowable uniform thickness variation is 293.8 nm. Smith & Welsh measured the relative temperature sensitivity of the piezoelectric coupling constant to be of the order of 1 × 10^−4^ °C^−1^ (Smith & Welsh, 1971 ▸), so temperature stabilization of approximately 10°C is required. Voltage control can compensate uniform changes in β or *h* and the temperature dependence of the coupling constant. However, nonuniform spatial variations in these parameters would require more detailed analysis and specification.

These estimates reveal the ranges appropriate for modern X-ray optical systems. Many optical elements would have looser specifications in practice. Optical elements designed for diffraction-limited performance would have tighter specifications.

## 7. Conclusion

As adaptive X-ray mirror technology matures, new applications become possible for beam shaping, steering, and wavefront control at fine length scales and at higher speeds. In this study, we present a general framework for mirror surface shaping that benefits from the accuracy and simplicity of lithographic patterning. We show through analytic derivation, supported by finite-element modeling, that shaping the electrode width along the length of the mirror controls the mirror curvature linearly when a voltage is applied.

This approach is particularly applicable to heat-treated lithium niobate mirror substrates because they behave inherently as monolithic bimorphs and do not require the addition of piezoceramic materials, by gluing, bonding, or growth. They offer a versatile and fabrication-friendly path toward compact, drift- and hysteresis-free adaptive X-ray mirrors.

Researchers familiar with wafer-thick substrates will recognize the significant challenges involved in achieving (and measuring) flat surfaces, much less curved surfaces with nanometre shape tolerances. Thin substrates are vulnerable to stresses related to processing, and deformation from mechanical mounting and gravity—effects that can overwhelm fine shape control. We have shown a mechanism for shape *changes*, while recognizing that successful implementations must address the factors that deform the mirror from its ideal shape at rest. We believe that it should be possible to customize electrode widths for specific, measured wafer deformations, or to adjust them after fabrication, to compensate for measured aberrations by adding or removing material.

Modeling demonstrations of uniform and variable-width electrodes set the stage for more advanced work and prototyping required to achieve ultra-high shape accuracy levels suitable for X-ray beamline applications. For example, the compound electrode concept illustrates how multiple optical functions can be realized on a single substrate using one applied voltage. In principle, this concept could be applied to the fine control of low-order aberrations, achieved with several narrow electrode channels arranged symmetrically.

## Figures and Tables

**Figure 7:**
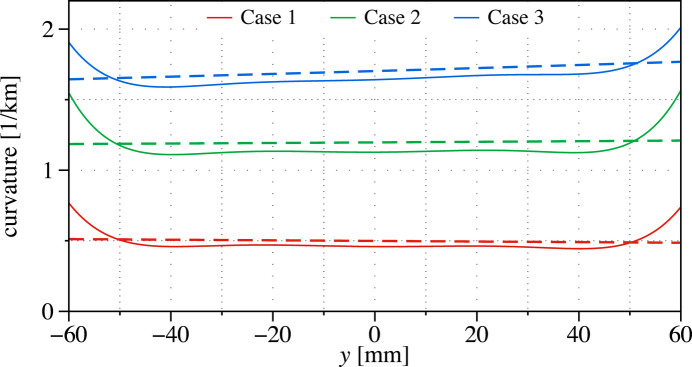
Comparison of the target curvatures (dashed lines) and those extracted from the FEA models (solid lines).
